# Awareness of Religious Leaders’ Fatwa and Willingness to Donate Organ

**Published:** 2015-11-01

**Authors:** M. Afzal Aghaee, M. Dehghani, M. Sadeghi, E. Khaleghi

**Affiliations:** 1 Department of Biostatistics, School of Health, Mashhad University of Medical Sciences, Mashhad, Iran; 2 Department of Epidemiology, School of Medicine. Shahroud University of Medical Sciences, Shahroud, Iran; 3 Department of Epidemiology, School of Public Health, Shahid Beheshti University of Medical Sciences, Tehran, Iran; 4 *Department of Organ Transplant, School of Medicine, Mashhad University of Medical Sciences, Mashhad, Iran*

**Keywords:** Knowledge, Tissue and organ procurement, Hospitals, religious, Islam, Consent forms

## Abstract

**Background:**

It is believed that religious leaders’ positive attitude towards organ donation can be an effective factor in Muslims’ inclination to donate organs.

**Objective:**

To assess the knowledge of freshmen students in Mashhad University of Medical Sciences about religious leaders’ *fatwa* on organ donation and its effect on their willingness to donate organs.

**Methods:**

This cross-sectional study was conducted in 2013 on 400 freshmen of various medical disciplines, selected using a simple random sampling in Mashhad, Iran. Data were collected by a valid and reliable researcher-made questionnaire. Data were analyzed by multiple logistic regression analysis.

**Results:**

41.5% of the students were aware of religious authorities’ views on organ donation and 55.6% were willing to donate organs. Participants’ main reasons for lack of willingness to donate organs included the fear of organ donation before the brain death is confirmed (52%), unwillingness to disfigure their body (51%), and belief in the burial of organs (50%). The willingness to organ donation for students who were aware of religious leaders opinion was more than twice more than those who were not (OR: 2.56, 95% CI: 1.75–4.52). Also, female gender, the Shia religion and awareness of the correct definition of brain death were associated factors affecting the desire to donate organs, although their effects were not statistically significant on regression model.

**Conclusion:**

A considerable proportion of students were not aware of the religious leaders’ *fatwa* on organ donation. The most important factor for the desire to donate organs was the awareness of religious leaders’ *fatwa*. Therefore, it seems necessary that religious leaders’ *fatwa* be known to all by appropriate methods.

## INTRODUCTION

Organ transplantation is of utmost importance for patients diagnosed with end-stage organ failure [1]. It was estimated that more than one million people round the globe receive an organ transplant annually [2]. In fact along with breakthroughs in the field of medicine and increased life expectancy, most countries in the world undergo an increasing need for organ transplantation [3, 4].

One of the most critical challenges of organ transplantation regards the scarce resources, inducing a huge gap between the number of potential recipients on transplant waiting list and the number of donor organs available [5, 6]. Grafts from cadaver are vastly considered the main supply. Probable legal, societal and lethal issues regarding the decision-making processes of brain death lead to controversy beyond medical forums. In other words, after the diagnosis of brain death, decease survivors adopt various approaches deriving from their religious and cultural backgrounds [7-10]. In religious contexts, human life is of high dignity and organ donation is approved [11-12]. A declaration with respect to the current affairs by Muslim competent authorities is called *fatwa*. From Islamic perspectives, the organ donation form cadaver is positively accepted without any restrictions [13-15].

Iran is among the countries where Islam is the dominant religion. Organ donation law in Iran was first passed in 2000 by Islamic Consultative Assembly. Reports had been demonstrated that the rate of cadaveric organs for transplantation in the country significantly increased during 2001-2010 [2]. Nevertheless, some studies confirmed that the majority of the public is unaware of the religious leaders’ attitude towards the matter [16-19]. Positive attitude (*fatwa*) on the part of religious authorities can vehemently impact the Muslims’ willingness on organ donation, which, in turn, will reduce the present gap. Most earlier attempts predominantly limited to donors’ religious views on organ transplantation [20-22]. Few studies have concerned with religious leaders’ sights on the allowance of organ transplantation following brain death and their subsequent potential effects on public tendencies. In this study, we aimed to elucidate the awareness of fresh entrants in Mashhad University of Medical Sciences on religious leaders’ views regarding the organ donation and assessing their impacts on the students’ willingness to donate organs.

## MATERIALS AND METHODS

This cross-sectional study was conducted in 2013 on 400 freshmen of various medical disciplines selected by simple random sampling method. They were first-year students and had not been exposed to medical education courses. We did not assess their level of religiosity before study. A researcher-made questionnaire was used to collect data. The questionnaire was developed based on reviewing all relevant and reliable documents and also experts’ views on the research subject. The questionnaire contained two parts of questions including demographic variables and individual’s inclination to donate theirs’ and their relatives’ organs after brain death, inclination to donate kidney to relatives, awareness of brain death definition, organ donation conditions, organs that can be donated, reasons for the reluctance to donate theirs’ and their relatives’ organs after brain death, organ donation membership cards and awareness of religious authorities’ views on organ donation. Before distributing the questionnaire, the study objectives were explained to the participants and after obtaining verbal informed consent the selected students entered into the study. Content validity (approved by the relevant experts) and reliability (using Cronbach’s α) of the questionnaire were 80% and 87%, respectively. Data were analyzed using SPSS^®^ for Windows^®^ ver 16. For univariate analysis of quantitative and qualitative variables, independent-sample *Student’s t* test and χ^2^ test were used. Multiple logistic regression analysis was used to determine factors influencing people’s inclination to donate organs. A p value <0.05 was considered statistically significant.

## RESULTS

Students’ response rate was 93.3%. Most participants were female (62.7%). The mean±SD age of participants was 19.4±2.3 years. All participants were Muslim, 74.4% were Shia and the rest were Sunni. Medical and nursing students comprised 38.8% of the participants; other participants were studying paramedics and health.

Only 23.4% of participants were aware of the full definition of brain death. For most participants (74.8%) having organ donation card was enough. Only 3% of participants believed that heirs’ consent is required for organ donation from brain-dead donors who have organ donation cards. A significant number of participants had the right information about organs that can be transplanted. Based on their responses, kidney (94.9%), heart (91.7%), cornea (83.7%) had the highest potential for organ transplantation and bone (39.7%) and pancreas (29.6%) had the lowest potential.

Fewer than half of the subjects (41.5%) were aware of the religious authorities’ views about organ donation (Table 1); most of them (88%) stated that religious leaders agree with organ donation from brain-dead patients; 55.6% of students who were aware of religious leaders’ positive view desired to donate organs, while only 30% of students who believed religious leaders do not agree with organ donation desired to donate organs. Only 3.3% of participants had an organ donation card. According to participants, a small percentage of their relatives (4%) had an organ donation card. About 6.1% of participants had a history of exposure to an individual with organ failure.

**Table 1:** Characteristics of study population

Variable	Willingness to donate organ		
	Yes (%)	No (%)	p value
Sex			
Male	46 (34.3)	88 (65.7)	0.18
Female	100 (43.1)	132 (56.9)	
Religion			
Sunni	5 (21.7)	18 (78.3)	0.08
Shiah	40 (41.1)	201 (58.9)	
Knowing the definition of brain death			
False (reference group)	81 (57.1)	132 (42.9)	0.60
Don’t know	10 (33.3)	20 (66.7)	
True	50 (38.0)	58 (62.0)	
Awareness of positive view of religious officials			
No (reference group)	65 (31.9)	139 (68.1)	<0.001
Yes	75 (55.6)	60 (44.4)	

Most participants (73.2%) agreed to donate their organs after brain death; however, fewer of them (40.1%) agreed to donate their relatives’ organs after brain death. The main reasons for participants’ uninterested to donate their own and their relatives’ organs included the fear of organ donation before brain death is confirmed (52%), unwillingness to disfigure their body (51%), belief in the burial of organs (50%), religious beliefs (39.7%) and lack of parents’ permission (35.6%).

In this study, the effect of factors such as gender, age, knowledge of brain death definition, organ donation law, knowledge of relevant* fatwas* on their interest to donate organs were examined by multiple logistic regression model. Logistic regression showed that only the effect of awareness from religious leaders’ views on organ donation was statistically significant on people’s desire to donate organs. In other words, the possibility of organs donation for students who were aware of religious leaders’ *fatwa* was 2.56 times as much as students who were not (Table 2). Also, female gender, the Shia religion and awareness of the correct definition of brain death were relevant factors affecting the desire to donate organs. This means that by controlling the effects of other variables under study, the possibility for the desire to donate organs in female students was 1.49 times as much as male students, in Shia Muslims was 1.68 times as much as Sunni Muslims, and in students who knew the correct definition of brain death was 1.25 times as much as students who were not aware of the correct definition. But the effect of these variables was not statistically significant in the multiple regression analysis. 

**Table 2:** Risk factors and odds for willingness to donate organs based on multiple logistic regression analysis

Variable	Crude			Adjusted	
	OR (95% CI)	p value		OR (95% CI)	p value
Age	1.02 (0.96–1.08)	0.57		1.03 (0.95–1.11)	0.49
Sex					
Male (reference group)	1.45 (0.93–2.25)	0.09		1.49 (0.89–2.39)	0.13
Female					
Religious					
Sunni (reference group)	2.51 (0.91–6.91)	0.08		1.68 (0.58–4.88)	0.34
Shiah					
Knowing definition of brain death					
False (reference group)					
Don’t know	0.82 (0.36–1.83)	0.62		0.98 (0.42–2.29)	0.96
True	1.41 (0.88–2.25)	0.15		1.25 (0.74–2.12)	0.41
Awareness of positive religious					
No (reference group)	2.67 (1.71–4.19)	<0.001		2.56 (1.75–4.52)	<0.001
Yes					

OR: Odds Ratio

CI: Confidence Interval

## DISCUSSION

The rate of organ donation in Muslim countries is lower than that in other countries [21]. In Iran, a Muslim country, while more than 15,000 brain deaths occur annually due to accidents, less than 10% of them are organ donors [26]. Despite existing laws on organ donation and religious leaders’ support, the low rate of organ donation could be due to insufficient awareness of religious leaders’ views on this issue. In this study, only 23.4% of participants were aware of the full definition of brain death (*ie*, irreversible loss of brain and brainstem function). In other words, most participants were not familiar with the brain death concept. The same findings are observed not only in similar studies in Iran but also in almost similar studies in other Muslim countries [26-28]. Participants’ awareness was low about those organs that can be transplanted (*eg*, skin, pancreas, bone and lung), which is confirmed by similar studies conducted in other countries [29, 30]. 

A larger proportion of participants did not know religious leaders’ views on organ donation. In other words, only 41% of them were aware of religious leaders’ *fatwa* on this subject. According to them, most religious leaders (88%) agree with organ donation from brain dead patients. Among those who knew religious leaders’ positive views, 55.6% desired to donate organs. In contrast, 30 participants who believed that religious leaders do not agree with organ donation desired to donate organs. Similar studies have different results about participants’ awareness of religious leaders’ views that the difference can be attributed to cultural and religious differences and participants’ characteristics [9, 31, 32]. For example, in Broomand, *et al*’s study, only 31% of participants were aware of religious leaders’ agreement with organ donation from brain dead patients and the desire to donate organs was 88% in the group that knew religious leaders’ positive views compared to 64% of those who believed that religious leaders do not agree with organ donation from brain dead patients [22]. In another study conducted in Saudi Arabia, more than two-thirds of participants were aware of religious leaders’ views about organ donation from living and non-living donors and inclination to donate organs in those who knew religious leaders’ positive views about organ donation compared to those who were not aware of this issue was 80% and 19%, respectively [33]. 

According to our findings, inclination to donate organs was more in people who had a correct perception of brain death concept compared to those who were not familiar with the concept. This difference was however not statistically significant. This finding is similar to the findings of similar studies conducted in other countries [34]. However, there is a significant statistical association between the knowledge of irreversibility of brain death and inclination to donate organs in studies conducted in Iran [22, 26, 28]. It seems that the difference in results is attributed to the type of question investigated; in this study our question was about brain death, whereas in similar studies the association between irreversibility of brain death and inclination to donate organs was assessed. In the current study and other similar studies from Iran, there is a gap between awareness of brain death concept and inclination to donate organs, so that more than 33% of those who were not familiar with brain death definition inclined to donate organs. The gap between knowledge and inclination to donate organs might be attributed to uncertainty of individual’s perception in brain death definition [35]. Although most participants were interested to donate organs [72.3%], a very small percentage of them (3%) had organ donation card. Although similar studies conducted in other Muslim countries and Iran also reported similar results [22, 23, 26, 28, 33], in some studies where the study population was medical staff, the number of people who had organ donation card was higher than that in other studies. This difference can be due to the effect of training received during the study and awareness of centers where organ donation cards were given [32, 36]. 

Students stated that the most important reason for lack of interest in organ donation was fear of organ donation before brain death is confirmed. This finding has been mentioned in most studies about organ donation [22, 37]. Another finding of this study was that female students were 49% more interested in organ donation than male students, although this difference was not statistically significant. However, the findings of other studies confirm the claim [22, 38]. In a study conducted on Nigerian students, however, opposite results were found, that is, Nigerian male students were more inclined to donate organs. These differences were probably attributed to participants’ cultural characteristics [34]. 

In conclusion, awareness of religious leaders’ *fatwa*, as the most important factor, has a significant effect on people’s inclination to donate organs. Students’ knowledge about religious leaders’ *fatwa* was rather low. Accordingly, the religious leaders’ views should be known to all by appropriate means. It seems necessary to train religious leaders to promote their awareness about organ donation in academic sessions. In this way we can promote knowledge of public in local, regional, and provincial lectures delivered by religious leaders. Future studies to assess the effectiveness of various methods for promoting community awareness about religious leaders’ *fatwa* and their positive attitude towards this subject are needed.
